# Supplementary material for: Feeding state-dependent neuropeptidergic modulation of reciprocally interconnected inhibitory neurons biases sensorimotor decisions in Drosophila
Source: Nat Commun. 2025 Sep 2;16:8198. doi: 10.1038/s41467-025-61805-y (PMC12405588; doi:10.1038/s41467-025-61805-y)
Supplement: Supplementary file 1 — Supplementary Information [file 41467_2025_61805_MOESM1_ESM.pdf]

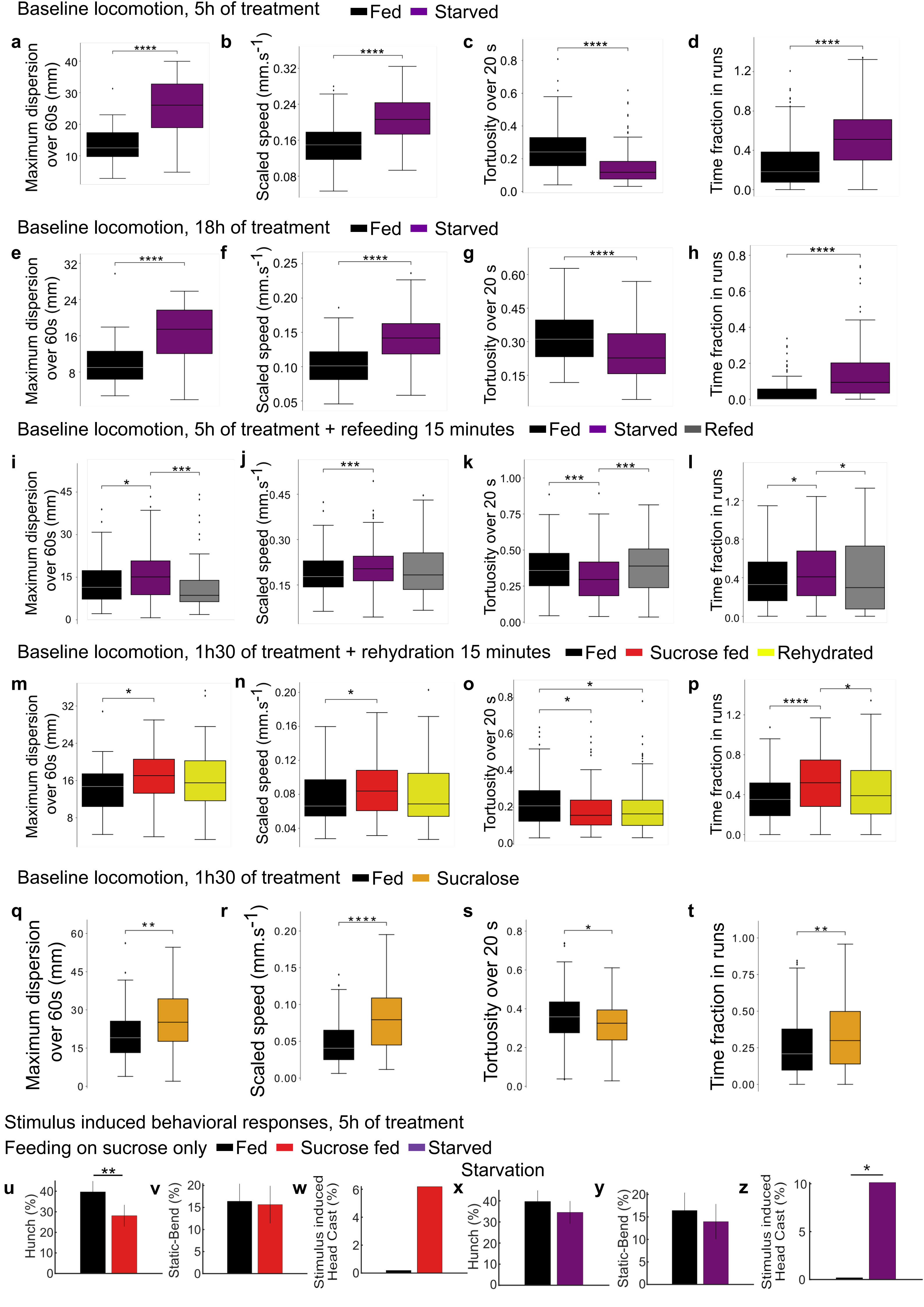

Baseline locomotion, 5h of treatment
Fed
Starved
a
b
c
d
Maximum dispersion over 60s (mm)
Scaled speed (mm.s-1)
Tortuosity over 20 s
Time fraction in runs
****
Baseline locomotion, 18h of treatment
e
f
g
h
Baseline locomotion, 5h of treatment + refeeding 15 minutes
Refed
i
j
k
l
*
***
Baseline locomotion, 1h30 of treatment + rehydration 15 minutes
Sucrose fed
Rehydrated
m
n
o
p
Baseline locomotion, 1h30 of treatment
Sucralose
q
r
s
t
**
Stimulus induced behavioral responses, 5h of treatment
Feeding on sucrose only
Starvation
u
v
w
x
y
z
Hunch (%)
Static-Bend (%)
Stimulus induced Head Cast (%)

Supplementary Fig. 1. Locomotion of food-deprived larvae. a-p Analysis of larval locomotion in different feeding states. **a-h** Larvae starved for 5h (**a-d**, fed n = 283 ; starved n = 309 larvae) or 18h (**e-h**, fed n = 173 ; starved = 139 larvae) disperse further, at a higher movement speed, allocating more time to crawling (time fraction in runs) with lower-tortuosity trajectories compared to larvae fed on a standard diet. **i-l** Refeeding larvae on standard food for 15 min after a period of 5h starvation rescues the normal locomotion as dispersion distance, time allocation to crawling and crawling speed are no longer significantly increased, nor the trajectories' tortuosity decreased compared to larvae fed on a standard diet (fed n = 244 starved n = 223 ; refed n = 160 larvae) **m-p** Rehydration by putting larvae for 15 minutes on water after 90 minutes of feeding of sucrose restores normal locomotion similar to larvae fed on standard food, since dispersion distance, time allocation to crawling and movement speed are no longer significantly increased, nor the trajectories' tortuosity decreased. **q-t** Feeding 23.2% sucralose (similar osmolarity as that of 20% sucrose) for 90 minutes to the larvae increases exploration (fed n = 193 ; sucralose n = 133 larvae) **u-z** Behavior in response to air-puff during the first five seconds upon stimulus onset upon 5h of sucrose feeding (**u-w**, fed n = 335 ; sucrose fed n = 281 larvae) and starvation (**x-z**, fed n = 335 ; starved n = 301 larvae). (Statistics: **a-t** two-sided Mann-Whitney test with Bonferroni correction; **u-v, x-y** Chi-square (one-sided) test; **w, z** Numerical simulation test; ****: $p < 0.0001$, ***: $p < 0.001$, **: $p < 0.01$, *: $p < 0.05$). The source data and p-values are provided in Source Data 1, 2, 3, and 5.

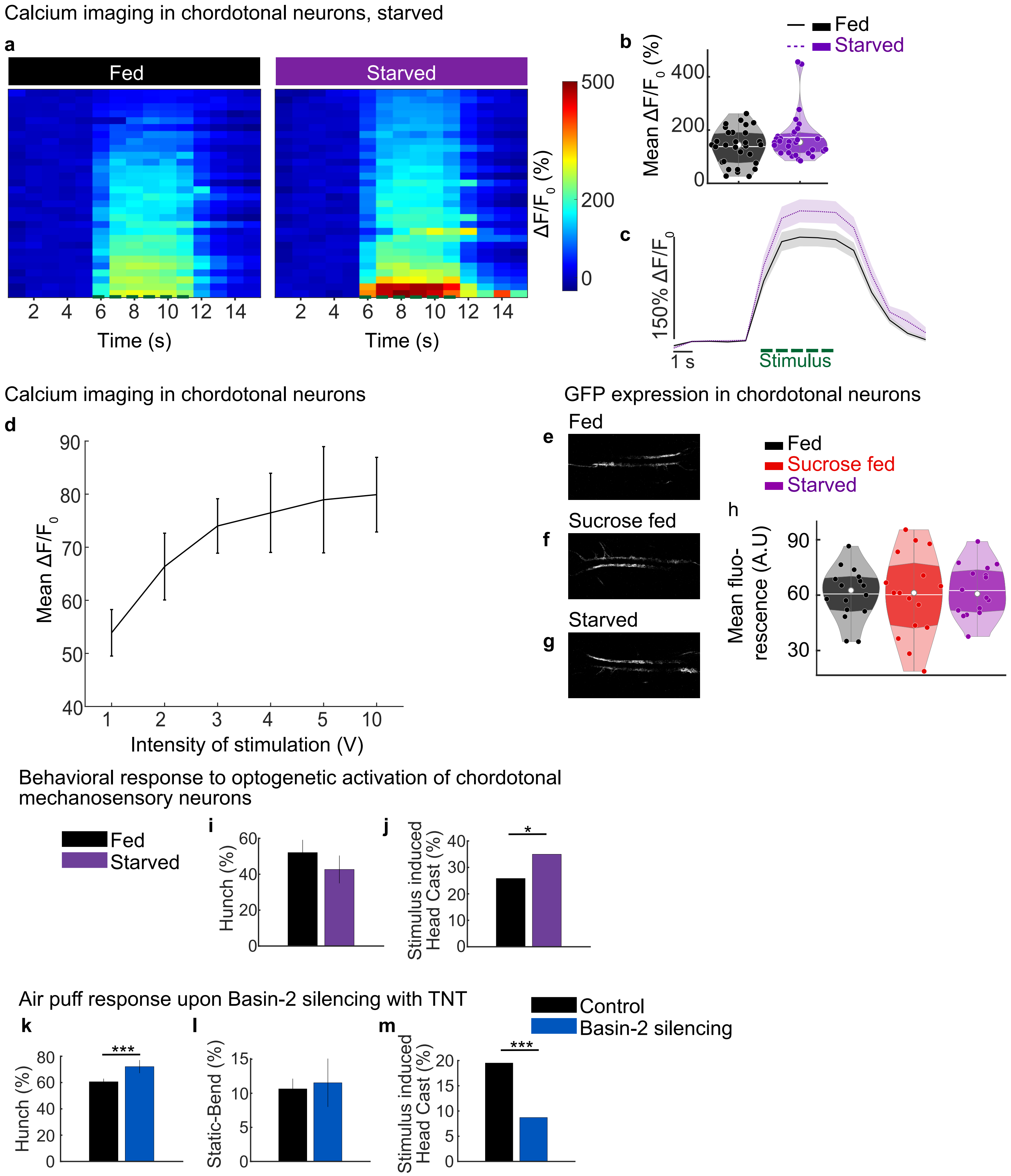
Calcium imaging in chordotonal neurons, starved
a
Fed
Starved
Time (s)
ΔF/F0 (%)
b
Mean ΔF/F0 (%)
Fed
Starved
c
150% ΔF/F0
1 s
Stimulus
Calcium imaging in chordotonal neurons
d
Mean ΔF/F0
Intensity of stimulation (V)
GFP expression in chordotonal neurons
e
Fed
f
Sucrose fed
g
Starved
h
Fed
Sucrose fed
Starved
Mean fluo-
rescence (A.U)
Behavioral response to optogenetic activation of chordotonal
mechanosensory neurons
Fed
Starved
i
Hunch (%)
j
Stimulus induced
Head Cast (%)
Air puff response upon Basin-2 silencing with TNT
Control
Basin-2 silencing
k
Hunch (%)
l
Static-Bend (%)
m
Stimulus induced
Head Cast (%)

Supplementary Fig. 2. Feeding state dependent bias in sensorimotor responses does not come from the modulation of sensory neurons. a-c Calcium responses to mechanical stimulations at 5V in chordotonal neurons in fed and starved larvae (R61D08-Gal4/UAS-GCaMP6s). **a** calcium responses of chordotonal neurons from different individuals fed on different diets. **b** calcium response averaged during the stimulus. White line represents the mean, white dot represents the median, colored dots with white edge represent individual data points. Stimulus-induced activity of chordotonal mechanosensory neurons is not significantly increased in starved animals as compared to larvae fed on a standard food (n = 10 larvae per condition, 3 trials per larva, two-tailed T-test, p-0.092.. **c** mean calcium trace of chordotonal neurons over time +/- SEM. The green dashed line corresponds to stimulus duration. **d** Calcium responses of chordotonal neurons to different intensities of mechanical stimulations, in larvae fed on standard food medium (n = 5 larvae, 1 trial for each intensity per larva). **e-h** GFP expressed in chordotonal neurons R61D08>GFP larvae fed on different food media: standard food (**e**), 20% sucrose (**f**) and water (**g**). **h** comparison of GFP fluorescence intensity as a proxy of transgene expression in R61D08>GFP larvae fed on different food media. White line represents the mean, white dot represents the median, colored dots with white edge represent individual data points. (n = 4 larvae per condition, 4 trials per larva). **i-j** Optogenetic activation of cho in starved larvae. Hunch (**i**) is cumulative probability during the first 2 seconds from stimulus onset. Bend (**j**) is the mean probability during the first 10 seconds from stimulus onset, corrected by 40 seconds of recording prior to the stimulus. Hunching and stimulus-induced Bending (fed n = 192 ; starved n = 157 larvae, p = 0.080 for hunch, 0.036 for bend). **k-m** Behavior in response to air-puff during the first five seconds upon stimulus onset for larvae in which Basin-2 was silenced using tetanus toxin (TNT) (GMR_SS00739>UAS-TNT, n = 312) compared to the control (n = 1625 larvae).(Statistics: **c-d, h** two-tailed T-test; **i, k-l** Chi-square (one-sided) test; **j, m** Numerical simulation test; ***: $p < 0.001$, **: $p < 0.01$, *: $p < 0.05$). The source data and p-values are provided in Source Data 2, 5, and 6.

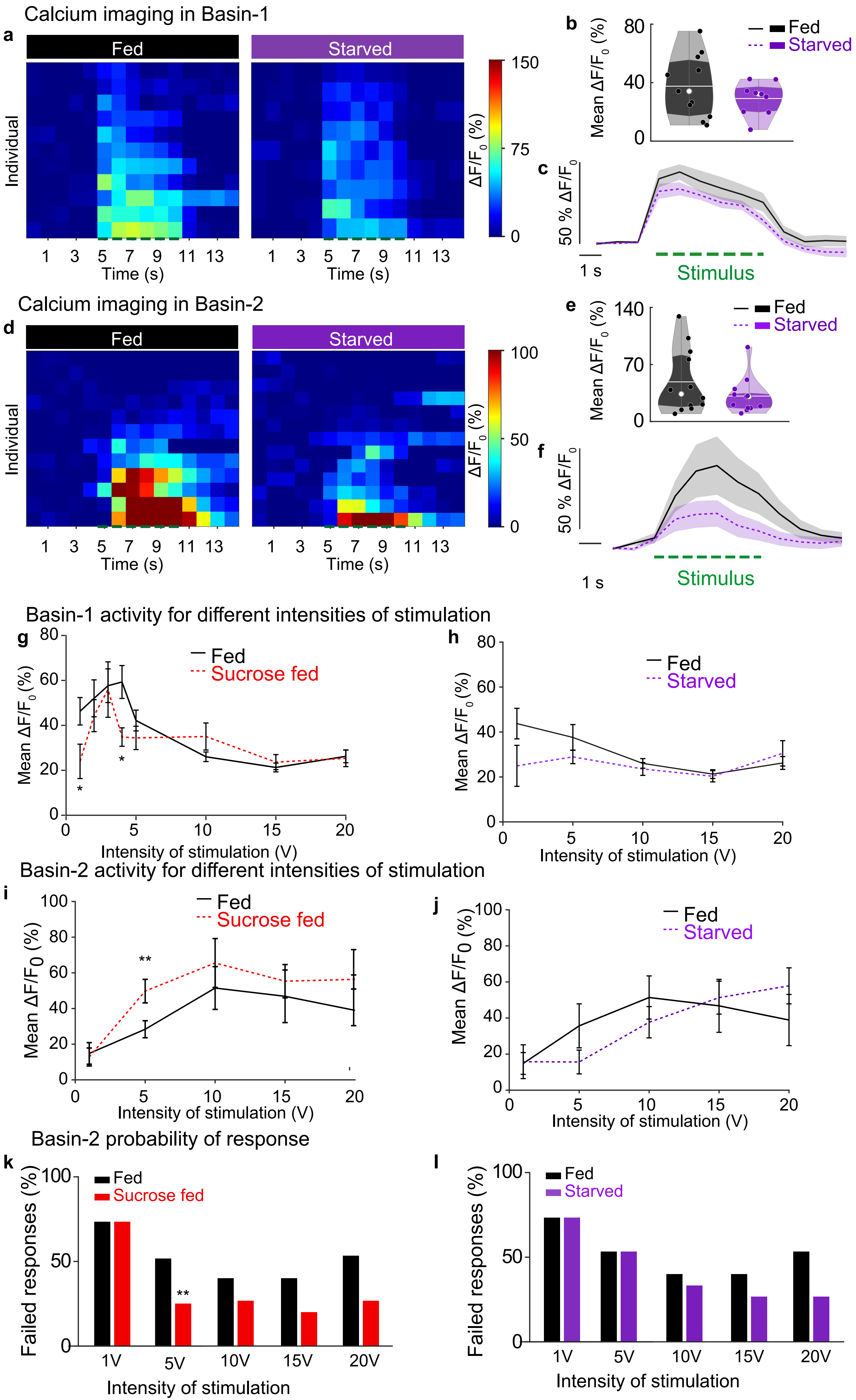
Calcium imaging in Basin-1
a
Fed
Starved
Individual
1 3 5 7 9 11 13
Time (s)
ΔF/F0 (%)
150
75
0
b
Mean ΔF/F0 (%)
80
40
0
Fed
Starved
c
50 % ΔF/F0
1 s
Stimulus
Calcium imaging in Basin-2
d
Fed
Starved
Individual
ΔF/F0 (%)
100
50
0
e
140
70
0
f
Basin-1 activity for different intensities of stimulation
g
Fed
Sucrose fed
Mean ΔF/F0 (%)
*
*
Intensity of stimulation (V)
h
Fed
Starved
Basin-2 activity for different intensities of stimulation
i
Fed
Sucrose fed
**
j
Fed
Starved
Basin-2 probability of response
k
Failed responses (%)
Fed
Sucrose fed
**
1V
5V
10V
15V
20V
Intensity of stimulation
l
Fed
Starved

Supplementary Fig. 3. Effect of starvation and different stimulus intensity on responses of projection neurons to mechanical stimulations. a-c Basin-1 calcium responses to mechanical stimulations in fed and starved larvae (20B01-lexA ; LexAop-GCaMP6s, UAS-CsChrimson-mCherry). **a** calcium responses of Basin-1 from different individuals fed on different diets. **b** mean calcium response averaged during the stimulus. White line represents the mean, white dot represents the median, colored dots with white edge represent individual data points. Stimulus-induced activity of Basin-1 neurons is similar in starved animals compared to larvae fed on a standard food(fed n = 11 ; starved n = 9 larvae, 1 trial per larva, two-tailed T-test p = 0.2892).**c** mean calcium trace of Basin-1 over time +/- SEM. The green dashed line corresponds to stimulus onset. **d-f** Basin-2 calcium responses to mechanical stimulations in different states (SS00739/UAS-GCaMP6s). **d** calcium responses of Basin-2 from fed and starved animals. **e** mean calcium response averaged during the stimulus. White line represents the mean, white dot represents the median, colored dots with white edge represent individual data points. Stimulus-induced activity of Basin-2 is not significantly decreased in starved animals compared to larvae fed on a standard food (fed n = 12 ; starved n = 13 larvae, 1 trial per larva, two-tailed T-test p = 0.1804).. **f** mean calcium trace of Basin-2 over time +/- SEM. The green dashed line corresponds to stimulus onset. **g-h** Basin-1 calcium responses for different intensities of stimulation, in larvae fed on standard food, on sucrose only (**g**) or completely starved larvae (**h**). Average neuronal activities over time for different intensities of stimulation are plotted. **i-j** Basin-2 calcium responses for different intensities of stimulation, in larvae fed on standard food, on sucrose only (**i**) or completely starved larvae (**j**). Averaged neuronal activity over time for different intensities of stimulation are plotted. **k-l** percentage of failed responses comparison. (Statistics: **b, e, g-j** two-tailed T-test; **k-l** Chi-square (one-sided) test; ***: p < 0.001, **: p < 0.01, *: p < 0.05). The source data and p-values are provided in Source Data 5 and 6.

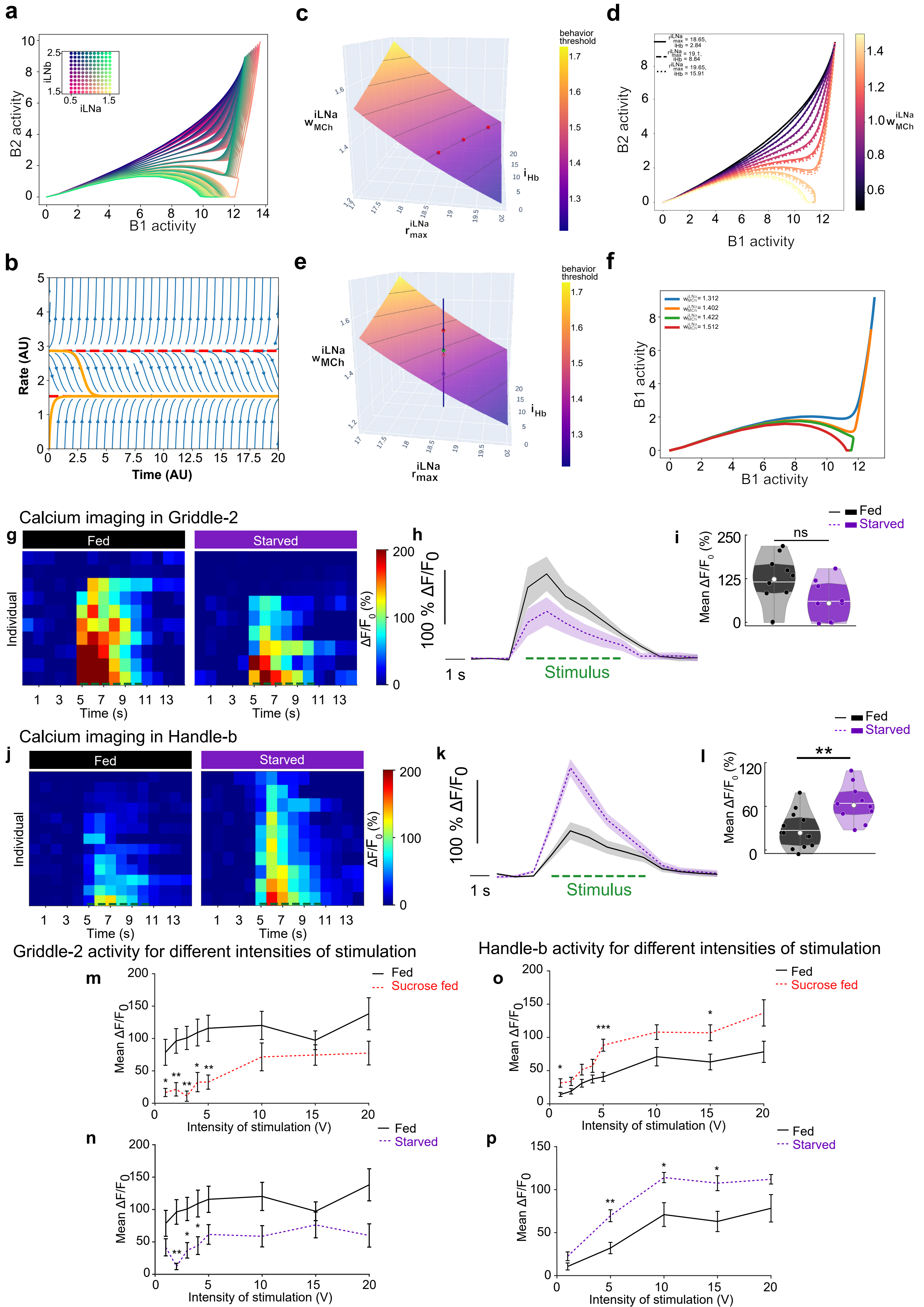
a
B2 activity
B1 activity
iLNb
iLNa
b
Rate (AU)
Time (AU)
c
behavior threshold
d
B2 activity
B1 activity
e
behavior threshold
f
B1 activity
B1 activity
Calcium imaging in Griddle-2
g
Fed
Starved
Individual
Time (s)
ΔF/F0 (%)
h
100 % ΔF/F0
1 s
Stimulus
Fed
Starved
i
ns
Mean ΔF/F0 (%)
Calcium imaging in Handle-b
j
Fed
Starved
Individual
Time (s)
ΔF/F0 (%)
k
100 % ΔF/F0
1 s
Stimulus
Fed
Starved
l
**
Mean ΔF/F0 (%)
Griddle-2 activity for different intensities of stimulation
m
Fed
Sucrose fed
Mean ΔF/F0
Intensity of stimulation (V)
n
Fed
Starved
Mean ΔF/F0
Intensity of stimulation (V)
Handle-b activity for different intensities of stimulation
o
Fed
Sucrose fed
Mean ΔF/F0
Intensity of stimulation (V)
p
Fed
Starved
Mean ΔF/F0
Intensity of stimulation (V)

Supplementary Fig. 4. Two interneuron subtypes are oppositely modulated by the feeding state. **a** State space trajectories of B1 and B2 Dynamics as a function of inputs to LNa and LNb neurons in a simple rate model (as in Jovanic *et al.,* 2016). **b** Dynamics of a single model neuron with self inhibition shows two equilibria, only one of which is stable **c-d** Level-set for threshold in combined model. **c** Points on lines at the intersection of the surface and horizontal planes represent sets of parameters with identical behavior response. **d** State-space trajectories of the models parameterized by the three points in **c** do not differ qualitatively **e-f** Combined model **e** Points on a vertical line correspond to the same model parameter, with different inputs to iLNa. Points above the surface converge to the monoactive state, while points below the surface converge to the coactive state. **f** Trajectories corresponding to the points in **e**, color-coded. **g-i** Griddle-2 calcium responses to mechanical stimulations in fed and starved larvae (SS00918/UAS-GCaMP6s). **g** calcium responses of Griddle-2 from different individuals fed on different diets. **h** mean calcium trace of Griddle-2 over time +/- SEM. The green dashed line corresponds to the stimulus. **i** mean calcium response averaged during the stimulus. White line represents the mean, white dot represents the median, colored dots with white edge represent individual data points. Stimulus-induced activity of Griddle-2 neurons is not significantly decreased in starved animals compared to larvae fed on a standard food (fed n = 10 ; starved n = 9 larvae, 1 trial per larva, two-tailed T-test, p = 0.0979) .. **j-l** Handle-b calcium responses to mechanical stimulations in different states (SS00888/UAS-GCaMP6s). **j** calcium responses of Handle-b from fed and starved animals. **k** mean calcium trace of Handle-b over time +/- SEM. The green dashed line corresponds to stimulus onset. **l** mean calcium response averaged during the stimulus. White line represents the mean, white dot represents the median, colored dots with white edge represent individual data points. Stimulus-induced activity of Handle-b is significantly increased in starved animals compared to larvae fed on a standard food (fed n = 13 ; starved n = 10 larvae, 1 trial per larva, two-tailed t-test p = 0.019).. **m-n** Griddle-2 calcium responses for different intensities of stimulation, in larvae fed on standard food, on sucrose only (**m**) and starved larvae (**n**). Average neuronal activities over time for different intensities of stimulation are plotted **o-p** Handle-b calcium responses for different intensities of stimulation, in larvae fed on standard food or on sucrose only (**o**) and starved larvae (**p**). Responses averaged during the stimulus for different intensities of stimulation are plotted. (Statistics: **i, l-p** two-tailed T-test; ***: p < 0.001, **: p < 0.01, *: p < 0.05). The source data and p-values are provided in Source Data 5 and 6.

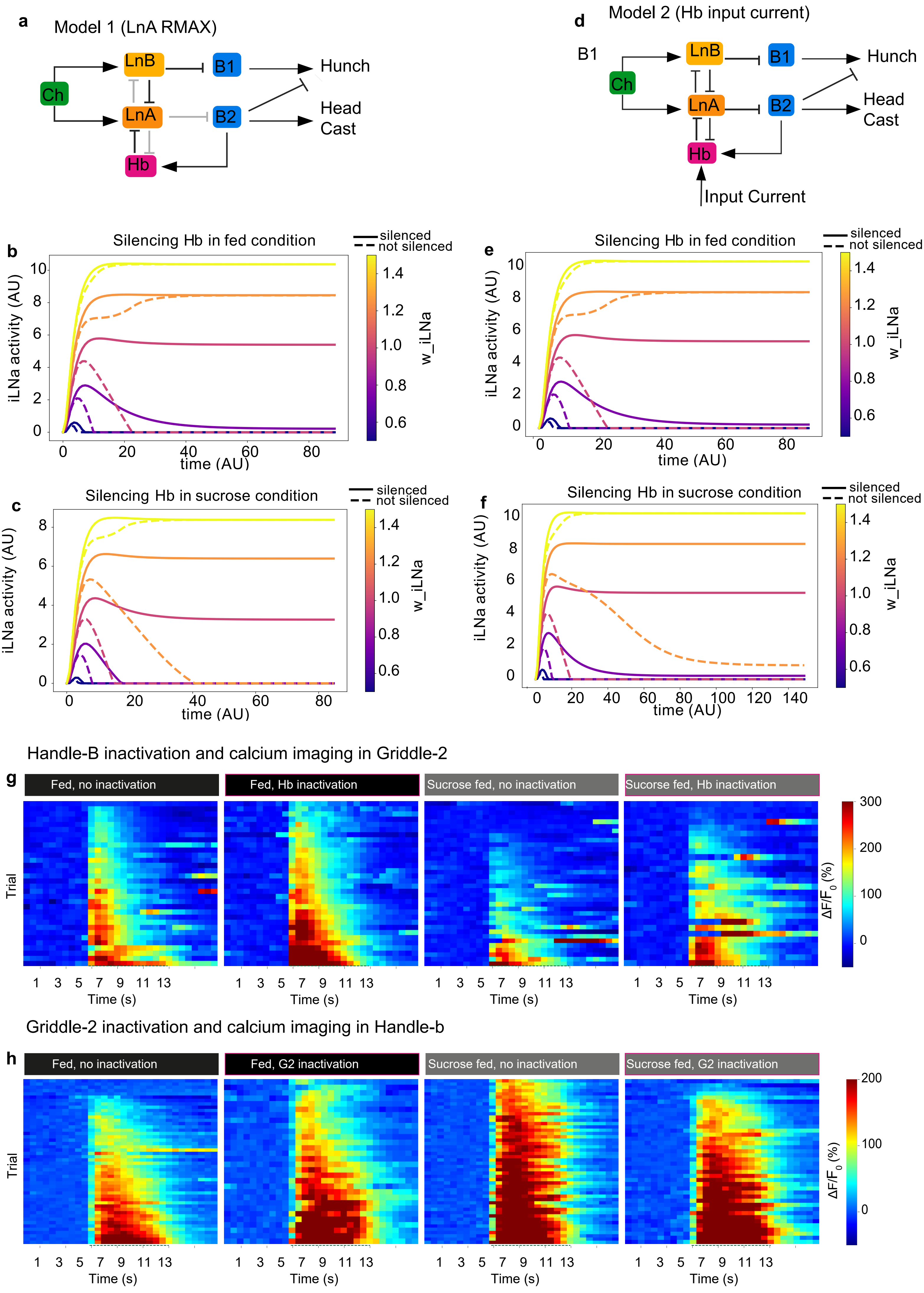
a
Model 1 (LnA RMAX)
Ch
LnB
LnA
Hb
B1
B2
Hunch
Head Cast
d
Model 2 (Hb input current)
B1
Input Current
b
Silencing Hb in fed condition
silenced
not silenced
iLNa activity (AU)
time (AU)
w_iLNa
c
Silencing Hb in sucrose condition
e
f
Handle-B inactivation and calcium imaging in Griddle-2
g
Fed, no inactivation
Fed, Hb inactivation
Sucrose fed, no inactivation
Sucorse fed, Hb inactivation
Trial
Time (s)
ΔF/F0 (%)
Griddle-2 inactivation and calcium imaging in Handle-b
h
Fed, G2 inactivation
Sucrose fed, G2 inactivation

Supplementary Fig. 5. Both G2 and Hb are modulated in a feeding-state dependent way . a-c Silencing Handle-b in model 1, where the sucrose state is modeled as a decrease in LNa max. **d-f** Silencing Handle-b in model 2 where the sucrose state is modeled as increase input in Handle-b. **g** Calcium responses in Griddle-2 with (55C05-LexA>LexAop-GCaMP6s SS00888-Gal4>UAS-TNT) or without Handle-b inactivation (55C05-LexA>LexAop-GCaMP6s +/UAS-TNT), in each trial of mechanosensory stimulation (fed no inactivation n = 8 ; fed with inactivation n = 8 ; sucrose fed no inactivation n = 9 ; sucrose fed with inactivation n = 7 larvae, 4 trials per larva). **h** Calcium responses in Handle with (55C05-LexA>LexAop-TNT 22E09-Gal4>UAS-GCaMP6s) or without Handle-b inactivation (+/LexAop-TNT 22E09-Gal4>UAS-GCaMP6s), in each trial of mechanosensory stimulation (fed no inactivation n = 10 ; fed with inactivation n = 6 ; sucrose fed no inactivation n = 10 ; sucrose fed with inactivation n = 40 larvae, 5 trials per larva). The source data and p-values are provided in Source Data 5 and 6.

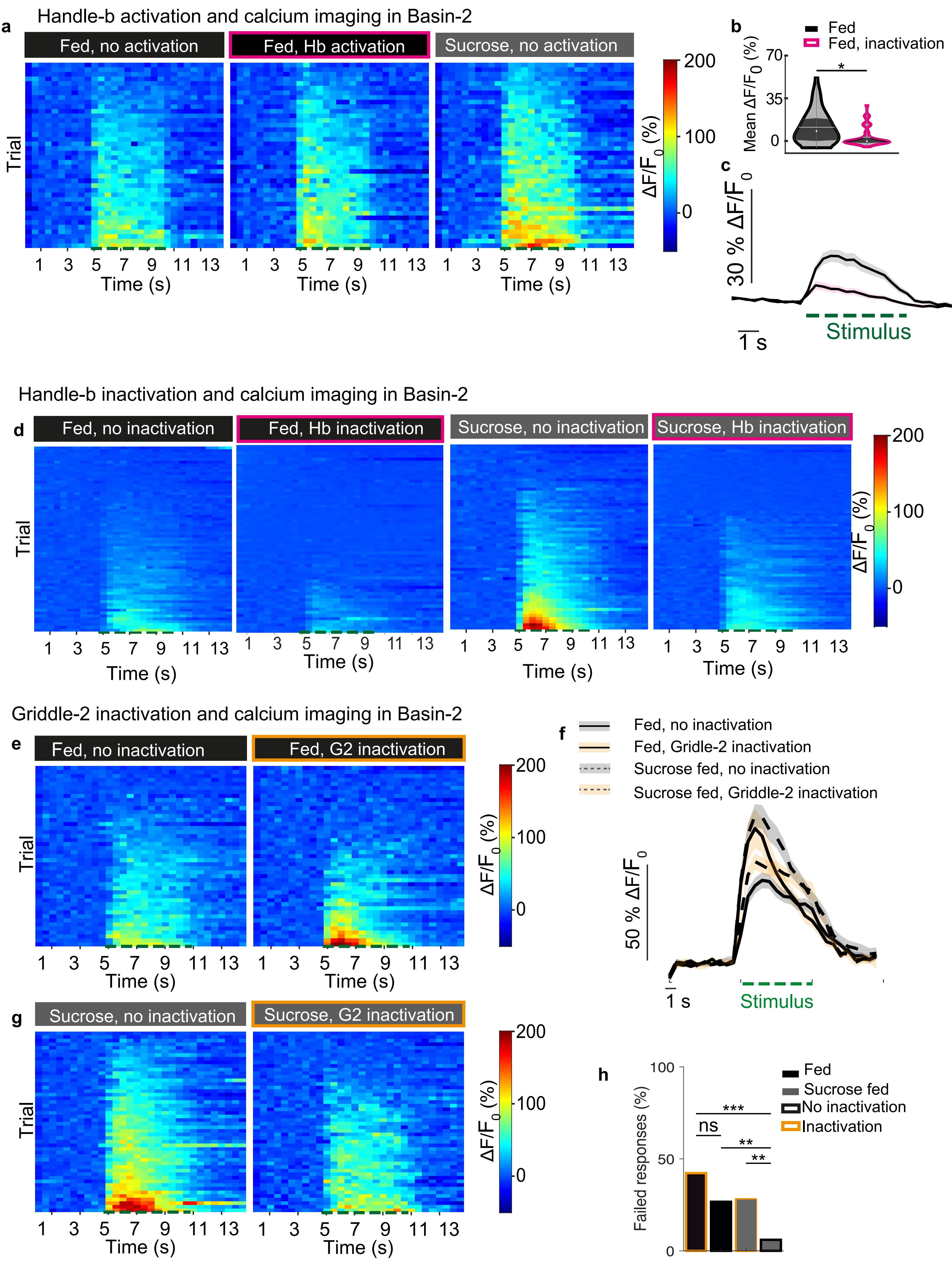
a
Handle-b activation and calcium imaging in Basin-2
Fed, no activation
Fed, Hb activation
Sucrose, no activation
Trial
1 3 5 7 9 11 13
Time (s)
ΔF/F0 (%)
200
100
0
b
Fed
Fed, inactivation
Mean ΔF/F0 (%)
70
35
0
*
c
30 % ΔF/F0
1 s
Stimulus
Handle-b inactivation and calcium imaging in Basin-2
d
Fed, no inactivation
Fed, Hb inactivation
Sucrose, no inactivation
Sucrose, Hb inactivation
Griddle-2 inactivation and calcium imaging in Basin-2
e
Fed, no inactivation
Fed, G2 inactivation
f
Fed, no inactivation
Fed, Gridle-2 inactivation
Sucrose fed, no inactivation
Sucrose fed, Griddle-2 inactivation
50 % ΔF/F0
g
Sucrose, no inactivation
Sucrose, G2 inactivation
h
Failed responses (%)
100
50
0
Fed
Sucrose fed
No inactivation
Inactivation
***
ns
**
**

Supplementary Fig. 6. Inhibitory interneurons are required for the feeding state dependent modulation of projection neurons. a Individual calcium responses in Basin-2 from individuals fed on different diets, with or without optogenetic activation of Handle-b (22E09-Gal4>UAS-CsChrimson::tdTomato 38H09-LexA>LexAop-GCaMP6s) during the first second of mechanical stimulus (fed no activation n = 8 ; fed with activation n = 8 ; sucrose fed no activation n = 7 larvae, 5 trials per larva). In all plots, the green dashed line corresponds to stimulus onset. **b** calcium response of Basin-2 upon Hb inactivation averaged during the stimulus. White line represents the mean, white dot represents the median with (38H09-LexA>LexAop-GCaMP6s 22E09-Gal4>UAS-TNT ; fed n = 12 ; sucrose fed n = 14 larvae) or without Handle-b inactivation (38H09-LexA>LexAop-GCaMP6s +/>UAS-TNT ; fed n = 13 ; sucrose fed n = 12 larvae). **c** mean calcium trace over time +/- SEM. The green dashed line corresponds to the stimulus. **d** Calcium responses in Basin-2 with (38H09-LexA>LexAop-GCaMP6s 22E09-Gal4>UAS-TNT ; fed n = 12 ; sucrose fed n = 14 larvae) or without (38H09-LexA>LexAop-GCaMP6s +/>UAS-TNT ; fed n = 13 ; sucrose fed n = 12 larvae) Handle-b inactivation in each trial of mechanosensory stimulation, in larvae fed on different diets. **e-h** Calcium responses in Basin-2 with (38H09-LexA>LexAop-GCaMP6s 55C05-Gal4>UAS-TNT ; fed n = 6 ; sucrose fed n = 8 larvae) or without (38H09-LexA>LexAop-GCaMP6s +/>UAS-TNT ; fed n = 10 ; sucrose fed n = 10 larvae) Griddle-2 inhibition, in each trial of mechanosensory stimulation, in larvae fed on standard food (**e**) or sucrose only (**g**). **f** mean calcium trace over time +/- SEM. The green dashed line corresponds to the stimulus. **h** percentage of failed responses. (Statistics: **b** one-way ANOVA with Tukey post-hoc test (two-sided); **h** Chi-square (one-sided) test; ***: $p < 0.001$, **: $p < 0.01$, *: $p < 0.05$). The source data and p-values are provided in Source Data 5 and 6.

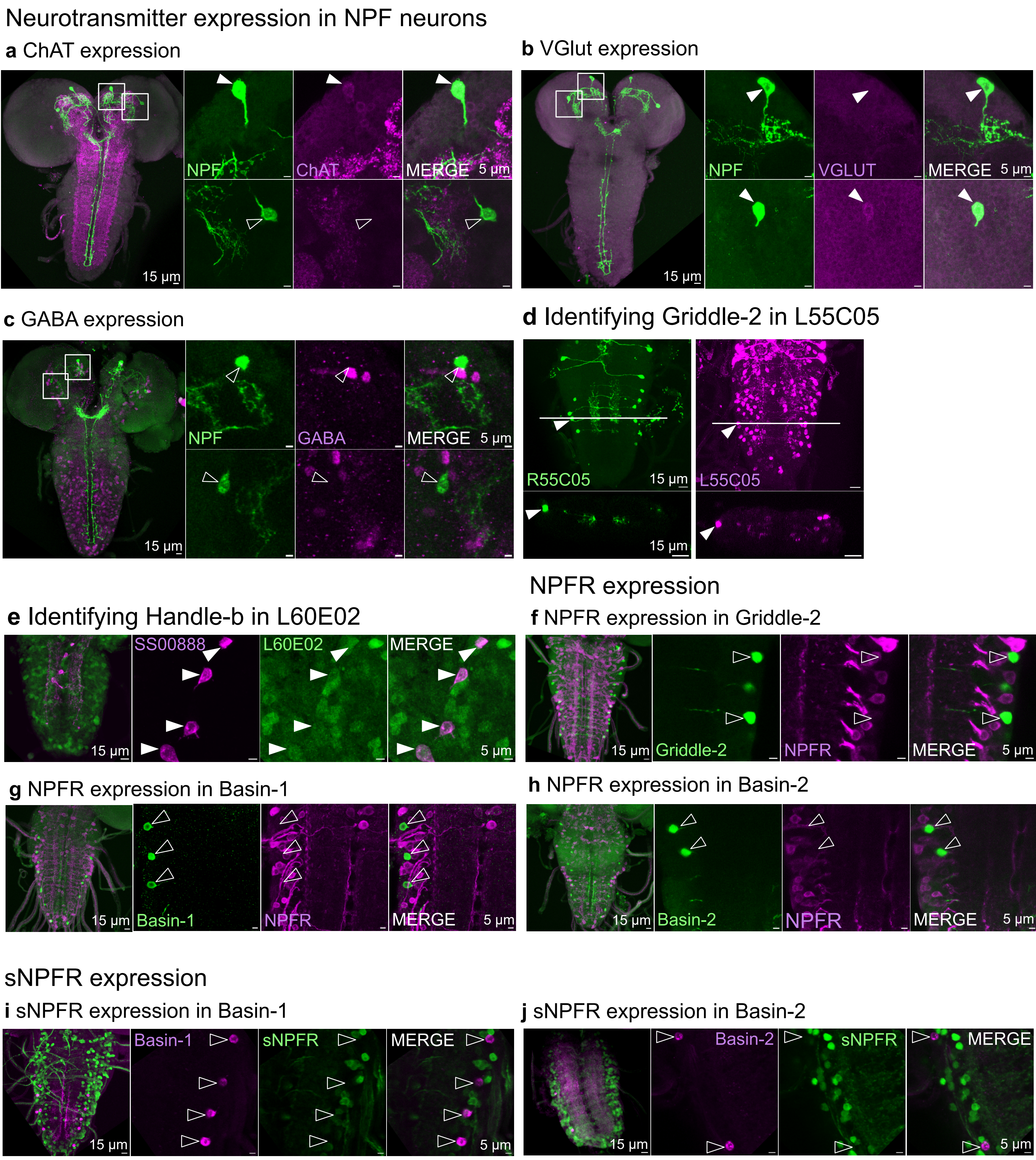

Neurotransmitter expression in NPF neurons
a ChAT expression
NPF
ChAT
MERGE
5 µm
15 µm
b VGlut expression
NPF
VGLUT
MERGE
5 µm
15 µm
c GABA expression
NPF
GABA
MERGE
5 µm
15 µm
d Identifying Griddle-2 in L55C05
R55C05
15 µm
L55C05
15 µm
NPFR expression
e Identifying Handle-b in L60E02
SS00888
L60E02
MERGE
15 µm
5 µm
f NPFR expression in Griddle-2
15 µm
Griddle-2
NPFR
MERGE
5 µm
g NPFR expression in Basin-1
15 µm
Basin-1
NPFR
MERGE
5 µm
h NPFR expression in Basin-2
15 µm
Basin-2
NPFR
MERGE
5 µm
sNPFR expression
i sNPFR expression in Basin-1
Basin-1
sNPFR
MERGE
15 µm
5 µm
j sNPFR expression in Basin-2
Basin-2
sNPFR
MERGE
15 µm
5 µm

Supplementary Fig. 7. Neurotransmitter identity of NPF neuron and NPF and sNPF receptor expression in neurons of the circuit. a-c Immunohistochemical labeling of NPF neuron neurotransmitters (NPF-Gal4>UAS-GFP) labels both pairs of NPF expressing neurons: dorsomedial (DM) and dorsolateral (DL). Brains were then stained with antibodies against either ChAT as a proxy of acetylcholine (**a**), V-Glut as a proxy of glutamate (**b**) and GABA (**c**). Co-localization shows expression of acetylcholine in the DL NPF neurons and glutamate in both pairs of NPF descending neurons. Both pairs were negative for GABA antibody labeling. **d** identifying griddle-2 in 55C05. The position of cell bodies and projections was compared between neurons in R55C05 (GAL4) (previously identified as Griddle-2) and L55C05 (LexA) that had a much broader expression pattern. **e** Identification of Handle-b in line L60E02. L60E02 is driving the expression of LexAop-GCaMP6s and GMR_SS00888 the expression of UAS-Chrimson-mCherry (60E02-LexA; UAS-Chrimson-mCherry, LexAop GCaMP6s>GMR_SS00888). Specific antibodies against GFP and mCherry were used to increase detection sensitivity. Co-localization of jRGECO1a and GCaMP6s show that the neuron with the cell body in the midline labeled by the 60E02-LexA is Handle-b. **f** Immunohistochemical labeling for NPFR in Griddle-2. UAS-GCaMP6s is expressed in Griddle-2 using the SS_TJ001 split-Gal4 line (green) and LexAop-jRGECO1a is expressed under the control of the NPFR promoter using a T2A-LexA construct (magenta). Antibodies against GFP and dsRed were used to increase detection sensitivity. No expression of NPFR could be detected in Griddle-2. **g-h** Basin-1 and -2 immunostaining for NPFR expression. UAS-GCaMP6s is expressed in Basin-1 or Basin-2 using the R20B01 or GMR_SS00739 driver lines (green) respectively and LexAop-jRGECO1a is expressed instead under the control of the NPFR transcript using a T2A-LexA construct (magenta). Antibodies against GFP and dsRed were used to increase detection sensitivity. No expression of NPFR could be detected in Basin-1 or -2. **i-j** Basin-1 and -2 immunostaining for sNPFR expression. LexAop-jRGECO1a is expressed in Basin-1 or Basin-2 respectively using the L20B01 or L38H09 lines (magenta) and UAS-GCaMP6s is expressed under the control of the sNPFR promoter using a T2A-Gal4 construct (green). Specific antibodies against GFP and dsRed were used to increase detection sensitivity. No expression of NPFR could be detected in Basin-1 or -2.

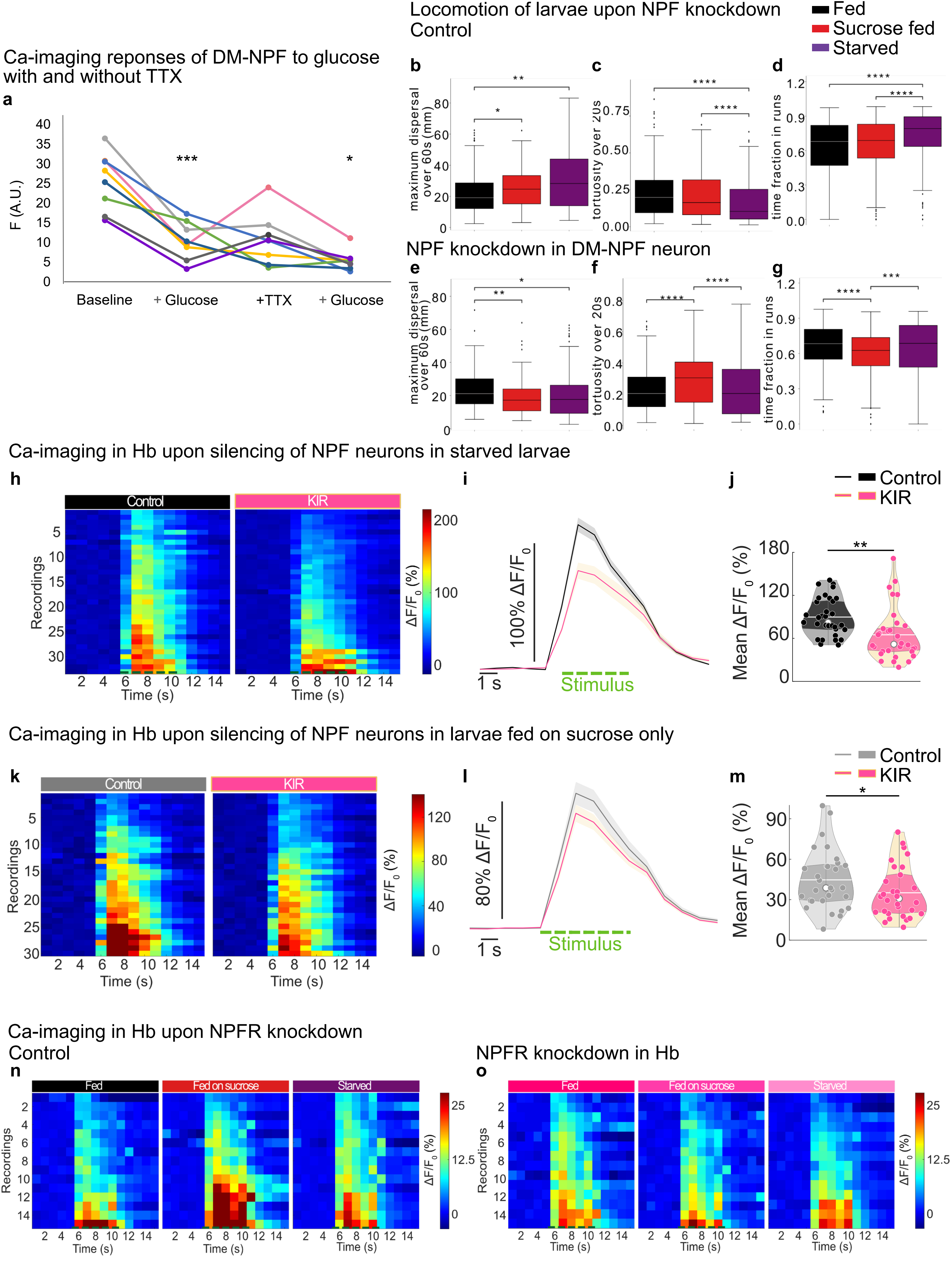

Ca-imaging reponses of DM-NPF to glucose with and without TTX
a
F (A.U.)
***
*
Baseline
+ Glucose
+TTX
+ Glucose
Locomotion of larvae upon NPF knockdown
Control
Fed
Sucrose fed
Starved
b
c
d
maximum dispersal over 60s (mm)
tortuosity over 20s
time fraction in runs
NPF knockdown in DM-NPF neuron
e
f
g
Ca-imaging in Hb upon silencing of NPF neurons in starved larvae
h
i
j
Control
KIR
Recordings
Time (s)
ΔF/F0 (%)
100% ΔF/F0
1 s
Stimulus
Mean ΔF/F0 (%)
Ca-imaging in Hb upon silencing of NPF neurons in larvae fed on sucrose only
k
l
m
80% ΔF/F0
Ca-imaging in Hb upon NPFR knockdown
Control
n
Fed
Fed on sucrose
Starved
NPFR knockdown in Hb
o

Supplementary Fig. 8. NPF regulates feeding state dependent changes in locomotion and Handle-b activity. a Changes in DM-NPF neuron activity upon glucose exposure with and without TTX. Calcium levels in NPF-Gal4; UAS-GCaMP6s larvae were measured following the application of 10.5% glucose, both before and after treatment with 20 μM TTX. NPF Calcium levels decreased significantly upon glucose application in both conditions, n = 8. Raw fluorescence is shown. **b-g** Analysis of larval locomotion in the absence of sensory stimulus. Top row shows control larvae (**b-d**, fed n = 405 ; sucrose fed n = 374 ; starved n = 348 larvae). Bottom row shows larvae with NPF knockdown in DM- NPF descending neurons (**e-g**, fed n = 359 ; sucrose fed n = 388 ; starved n = 327 larvae). NPF knockdown in DM-NPF descending neuron abolishes differences in locomotion mean between larvae in different states; it abolishes the difference in trajectory tortuosity, time allocation in exploration (time fraction in runs), and dispersal between larvae fed on standard food and starved larvae. In larvae fed on sucrose the increase in exploration compared to larvae fed on standard food was also abolished. **h-j** Calcium responses in Handle-b with (UAS-GCaMP6s; 22E09-Gal4, NPF-LexA>LexAop-KIR) or without (UAS-GCaMP6s; 22E09-Gal4, NPF-LexA;+) NPF neurons silencing in starved larvae. **h** calcium responses of Handle-b, in each trial of mechanosensory stimulation. **i** mean calcium trace of Handle-b over time +/- SEM. The green dashed line corresponds to the stimulus. **j** mean calcium response averaged during the stimulus. White line represents the mean, white dot represents the median, colored dots with white edge represent individual data points. Stimulus-induced activity of Handle-b is decreased upon Coaster silencing (n= 11 larvae per condition) . **k-m** Calcium responses in Handle-b with (UAS-GCaMP6s; 22E09-Gal4, NPF-LexA>LexAop-KIR) or without (UAS-GCaMP6s; 22E09-Gal4, NPF-LexA;+) NPF neurons silencing in larvae fed on sucrose only. **k** calcium responses of Handle-b, in each trial of mechanosensory stimulation. **l** mean calcium trace of Handle-b over time +/- SEM. The green dashed line corresponds to the stimulus. **m** mean calcium response during the first second of stimulus. White line represents the mean, white dot represents the median, colored dots with white edge represent individual data points. Stimulus-induced activity of Handle-b is decreased upon NPF neurons silencing. **n-o** Calcium responses in Handle-b upon NPFR knockdown (GMR_SS00888>UAS-NPFR-RNAi; UAS-GCaMP6s) compared to a control (GMR_SS00888>UAS-GCaMP6s). **n** calcium responses of Handle-b in control larvae fed, fed on sucrose or starved, in each trial of mechanosensory stimulation. **o** calcium responses of Handle-b upon NPFR knockdown in larvae fed, fed on sucrose or starved, in each trial of mechanosensory stimulation. (Statistics: **a, j** two-tailed T-test; **b-g** two-sided Mann-Whitney test with Bonferroni correction; **m** one-sided Mann-Whitney test; ****: p < 0.0001, ***: p < 0.001, **: p < 0.01, *: p < 0.05). The source data and p-values are provided in Source Data 1, 5, and 6.

Two-dimensional representation of Control and NPFR knockdown behavioral data with supervised UMAP

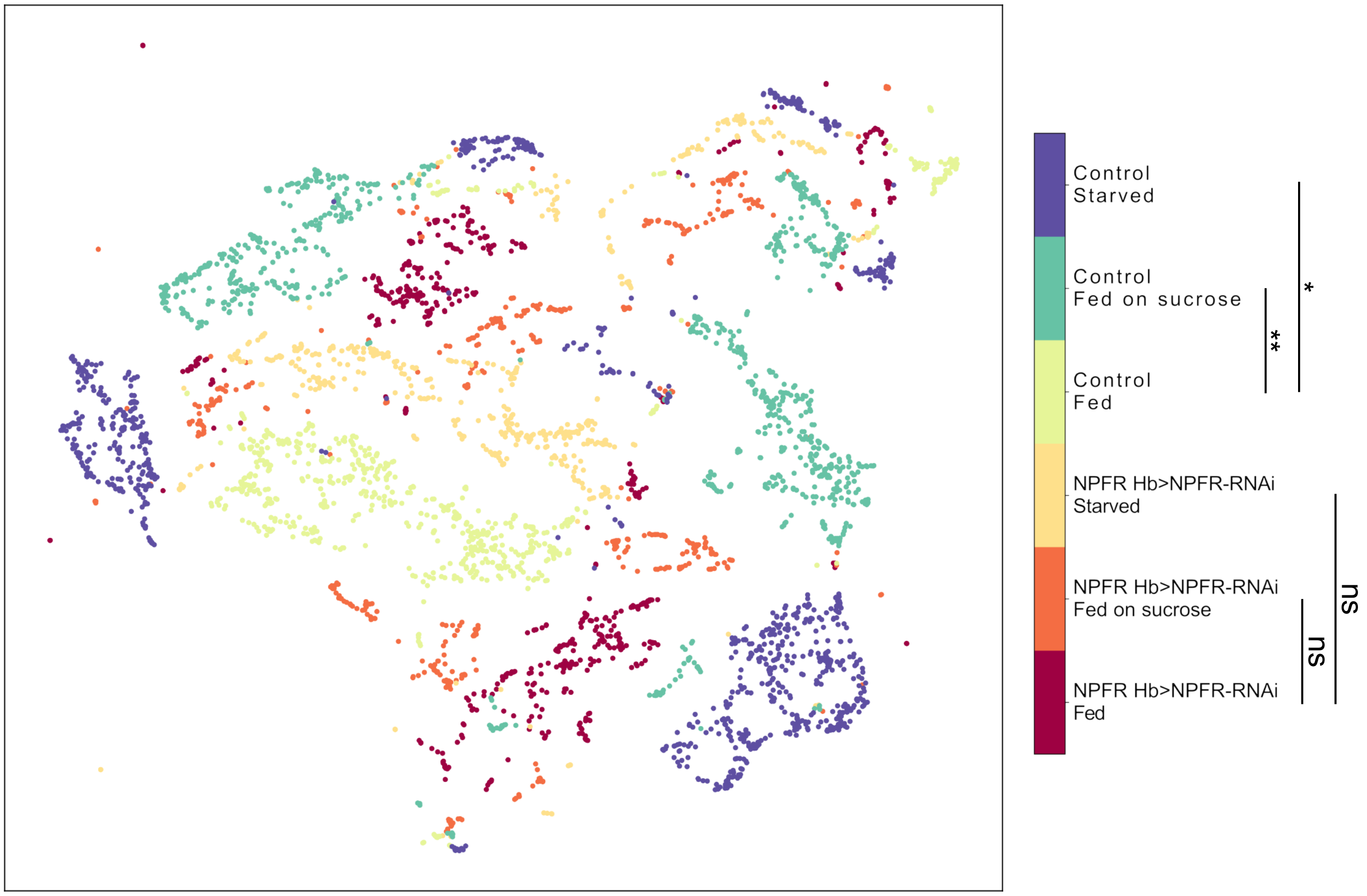

Supplementary Fig. 9. Role of NPF signaling in the feeding state-dependent modulation of larval behavioral response to an air puff. 2D representation of NPFR knockdown behavioral data with supervised UMAP, Each larva is represented by 5 time points in the 60-65 second time window. Control (SS00888>w1118; fed n = 138 ; sucrose fed n = 169 ; starved n = 189 larvae). Fed larvae are in the center of the space with sucrose fed and starved larvae occupying the periphery. (Maximum Mean Discrepancy test with Bonferroni correction, ***: $p < 0.001$, **: $p < 0.01$, *: $p < 0.05$). The p-values are provided in Source Data 5.

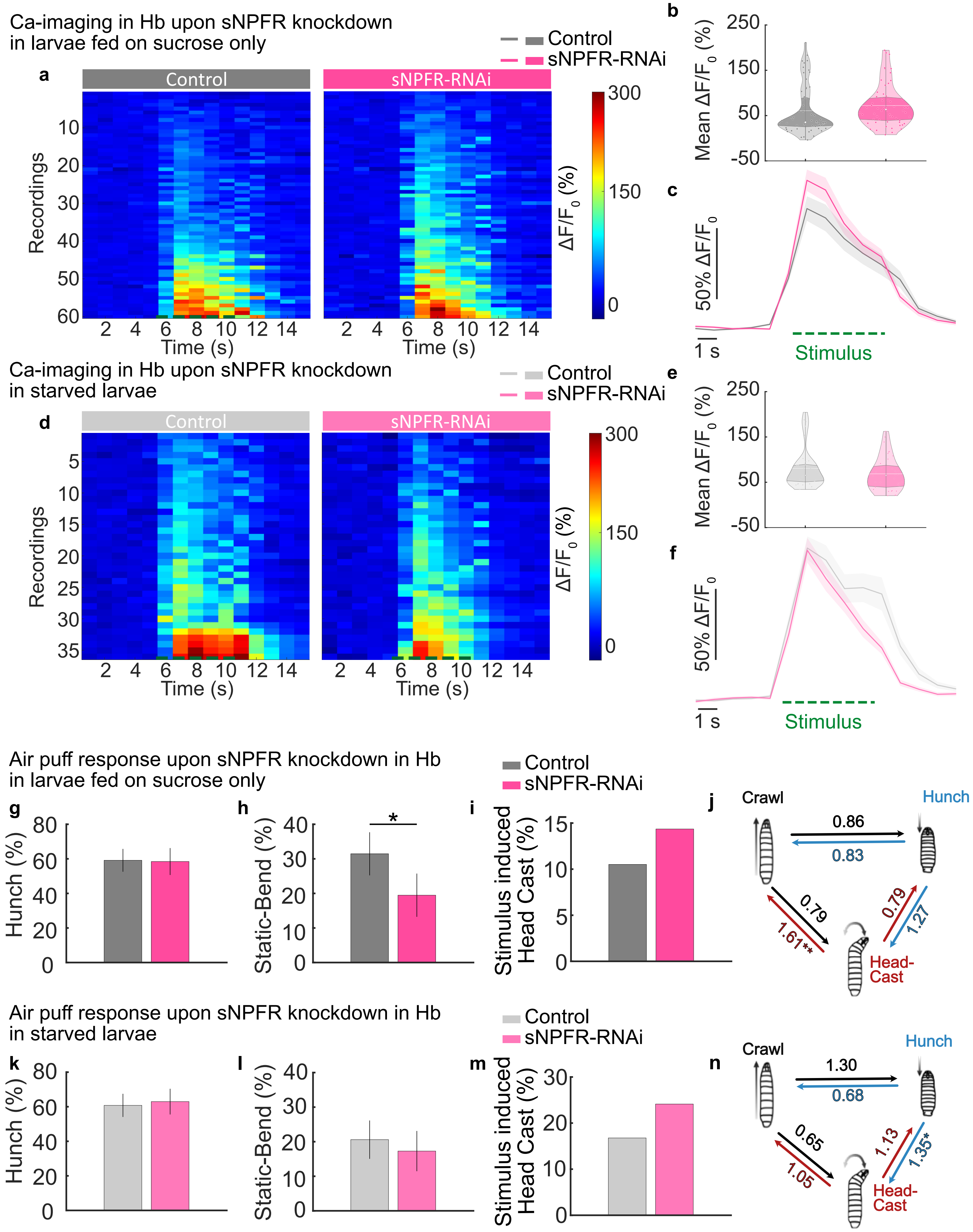

Ca-imaging in Hb upon sNPFR knockdown in larvae fed on sucrose only
Control
sNPFR-RNAi
a
b
c
Recordings
Time (s)
ΔF/F0 (%)
Mean ΔF/F0 (%)
50% ΔF/F0
1 s
Stimulus
Ca-imaging in Hb upon sNPFR knockdown in starved larvae
d
e
f
Air puff response upon sNPFR knockdown in Hb in larvae fed on sucrose only
g
h
i
j
Hunch (%)
Static-Bend (%)
Stimulus induced Head Cast (%)
Crawl
Hunch
Head-Cast
0.86
0.83
0.79
1.61**
0.79
1.27
Air puff response upon sNPFR knockdown in Hb in starved larvae
k
l
m
n
1.30
0.68
0.65
1.05
1.13
1.35*

Supplementary Fig. 10. sNPFR knockdown does not impact Handle-b responses in larvae fed on sucrose or starved larvae. a-f Calcium responses in Handle-b sNPFR knockdown in Handle-b (GMR_SS00888>UAS-GCaMP6s; UAS-sNPFR-RNAi) compared to the control (GMR_SS00888>UAS-GCaMP6s) in larvae fed on sucrose only (**a-c,** n = 15 larvae per genotype, 4 trials per larva) or starved (**d-f,** n = 9 larvae per genotype, 4 trials per larva). **a,d** calcium responses of Handle-b, in each trial of mechanosensory stimulation. **b,e** response averaged during the stimulus. White line represents the mean, white dot represents the median, colored dots with white edge represent individual data points. sNPFR knockdown does not influence the stimulus-induced activity of Handle-b in larvae fed on sucrose only or starved. **c,f** mean calcium trace of Handle-b over time +/- SEM. The green dashed line corresponds to stimulus onset. **g-i** Behavior in response to air-puff during the first five seconds upon stimulus onset for larvae fed on sucrose in which sNPFR was knocked down in Handle-b neurons (SS00888>sNPFR-RNAi, n = 154 larvae) compared to the control (n = 213 larvae). **j** Behavioral transitions over the first ten seconds of stimulation. **k-m** Behavior in response to air-puff during the first five seconds upon stimulus onset for starved larvae in which sNPFR was knocked down in Handle-b neurons (SS00888>sNPFR-RNAi, n = 162 larvae) compared to the control (n = 204 larvae). **n** Behavioral transitions over the first ten seconds of stimulation. (Statistics: **b, e** two-tailed T-test; **g-h, k-l** Chi-square (one-sided) test; **i, m** Numerical simulation test; **j, n** Maximum likelihood test (one-sided, chi-square approximation); ***: $p < 0.001$, **: $p < 0.01$, *: $p < 0.05$). The source data and p-values are provided in Source Data 2, 3, 5, and 6.

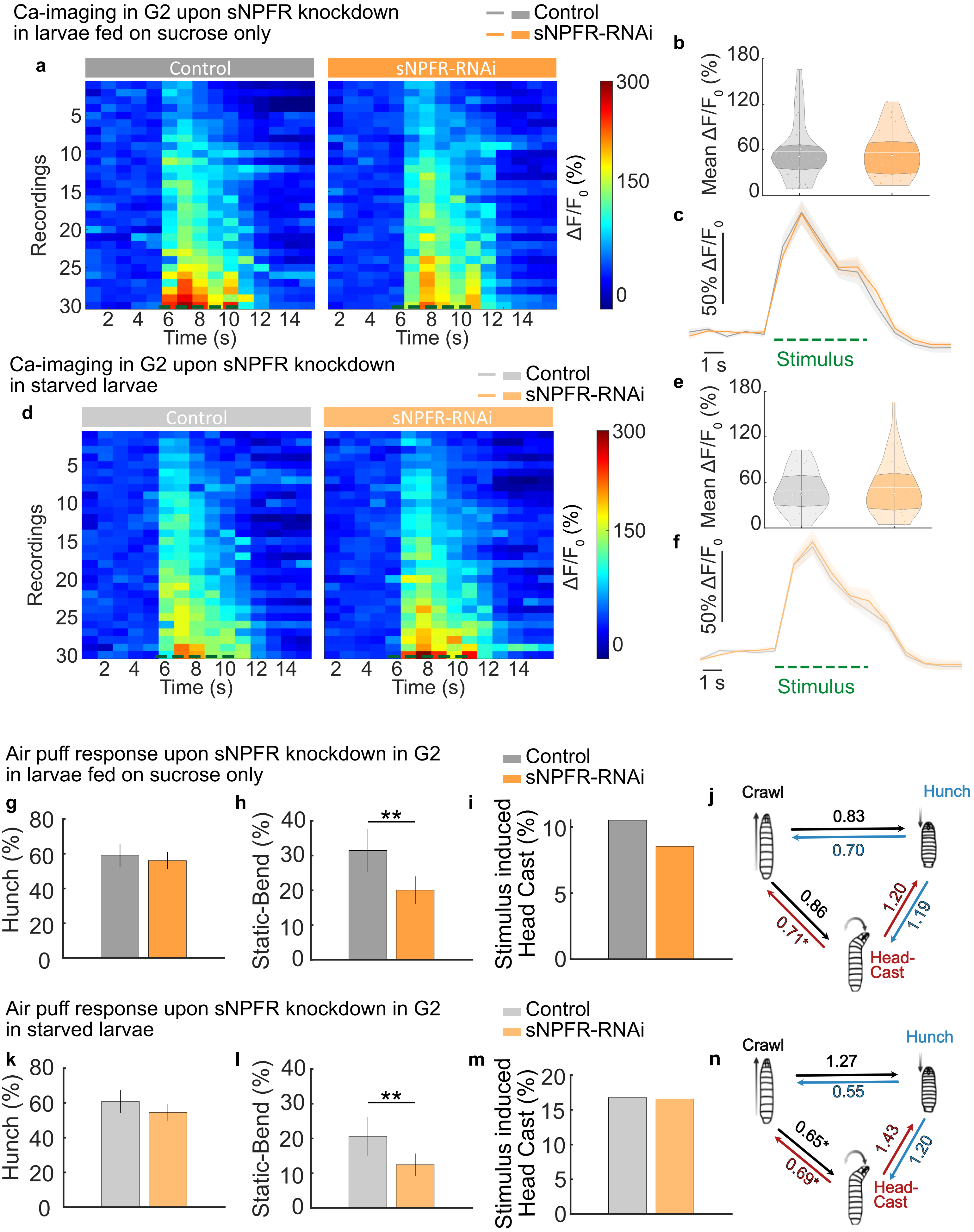
Ca-imaging in G2 upon sNPFR knockdown in larvae fed on sucrose only
Control
sNPFR-RNAi
a
Recordings
Time (s)
ΔF/F0 (%)
b
Mean ΔF/F0 (%)
c
50% ΔF/F0
1 s
Stimulus
Ca-imaging in G2 upon sNPFR knockdown in starved larvae
d
e
f
Air puff response upon sNPFR knockdown in G2 in larvae fed on sucrose only
g
Hunch (%)
h
Static-Bend (%)
**
i
Stimulus induced Head Cast (%)
j
Crawl
Hunch
Head-Cast
0.83
0.70
0.86
0.71*
1.20
1.19
Air puff response upon sNPFR knockdown in G2 in starved larvae
k
l
m
n
1.27
0.55
0.65*
0.69*
1.43
1.20

Supplementary Fig. 11. sNPFR knockdown does not impact Griddle-2 responses in larvae fed on sucrose or starved larvae. a-f Calcium responses in Griddle-2 with (SS_TJ001>UAS-GCaMP6s; UAS-sNPFR-RNAi) or without (SS_TJ001>UAS-GCaMP6s) sNPFR knockdown in Griddle-2 in larvae fed on sucrose only (**a-c**) or starved (**d-f**). **a,d** calcium responses of Griddle-2, for each trial. **b,e** mean calcium response averaged during the stimulus. White line represents the mean, white dot represents the median, colored dots with white edge represent individual data points. sNPFR knockdown does not influence the stimulus-induced activity of Griddle-2 in larvae fed on sucrose only or starved (n = 10 larvae per genotype and condition, 3 trials per larva). **c,f** mean calcium trace of Griddle-2 over time +/- SEM. The green dashed line corresponds to stimulus onset. **g-i** Behavior in response to air-puff during the first five seconds upon stimulus onset for larvae fed on sucrose in which sNPFR was knocked down in Griddle-2 neurons (SS_TJ001>sNPFR-RNAi, n = 394 larvae) compared to the control (n = 213 larvae). **j** Behavioral transitions over the first ten seconds of stimulation. **k-m** Behavior in response to air-puff during the first five seconds upon stimulus onset for starved larvae in which sNPFR was knocked down in Griddle-2 neurons (SS_TJ001>sNPFR-RNAi, n = 409 larvae) compared to the control (n = 204 larvae). **n** Behavioral transitions over the first ten seconds of stimulation. (Statistics: **b, e** two-tailed T-test; **g-h, k-l** Chi-square (one-sided) test; **i, m** Numerical simulation test; **j, n** Maximum likelihood test (one-sided, chi-square approximation); ***: $p < 0.001$, **: $p < 0.01$, *: $p < 0.05$). The source data and p-values are provided in Source Data 2, 3, 5, and 6.

Supplementary Method 1 Resource Table

	REAGENT or RESOURCE	SOURCE	IDENTIFIER (Official)
**Experimental Models: Organisms/Strains**	**Fly Stocks**		
Drosophila melanogaster	w1118(5905); +; pJFRC12-10XUAS-IVS-myr::GFP in attP2	Bloomington	32197
	CantonS		
	y w;;attP2	Pfeiffer et al, 2008, 10	Pfeiffer et al, 2008, 10
	y w; attP40; attP2	Pfeiffer et al, 2010	Pfeiffer et al, 2010
	w+; UAS-TNTe	Sweeney et al, 1995	Sweeney et al, 1995
	20XUAS-CsChrimson-mVenus trafficked in attP18	Bloomington	55134
	w[1118]; P{y[+t7.7] w[+mC]=GMR61D08-GAL4}attP2	Bloomington	39272
	w[1118]; CG10342-RNAi	VDRC	108772
	y[1] w[*]; P{w[+mC]=NPF-GAL4.1}2	Bloomington	25681
	SS01635 (R11H10-p65.AD}attP40;R10H10-GAL4.DBD}attP2/)	Gift from Marta Zlatic, Meissner et al, 2024	
	P{y[+t7.7] w[+mC]=R55C05-p65.AD}attP40/Cyo tb ; P{y[+t7.7] w[+mC]=R34G01-GAL4.DBD}attP2/Tm6 tb sb	This paper	
	w[1118]; P{y[+t7.7] w[+mC]=R82F03-p65.AD}attP40; P{y[+t7.7] w[+mC]=R34G01-GAL4.DBD}attP2	Bloomington	86674
	w[1118]; P{y[+t7.7] w[+mC]=R55C05-p65.AD}attP40	Bloomington	68650
	SS00888	Jovanic et al, 2016	
	LK-GAL4 [ChII]	gift from P. Herrero, Madrid, Spain	
	w[1118]; CG1174-RNAi	VDRC	107663
	w[1118]; empty RNAi-TK	VDRC	60000
	w-;LexAop Kir (VIE260B);	Feng et al, 2014	
	w[*]; P{w[+mC]=Gr43a-GAL4.0.5}9; Dr[1]/TM3, Sb[1]	Bloomington	57636

Supplementary Method 1 Resource Table

	w[1118]; P{y[+t7.7] w[+mC]=GMR20B01-GAL4}attP2	Bloomington	48877
	SS00739	Jovanic et al, 2016	
	SS00918	Jovanic et al, 2016	
	pGP-JFRC7-20XUAS-IVS-GCaMP6s 15.641 in VK00005	Bloomington	42749
	w[1118]; P{y[+t7.7] w[+mC]=GMR61D08-lexA}attP40	Bloomington	52722
	w[1118]; P{y[+t7.7] w[+mC]=GMR38H09-lexA}attP40	Bloomington	54102
	w*; 38H09-LexA (AttP40) ; 20xUAS-CsChrimson-mCherry-trafficked in su(Hw)attP1 (3015633), 13xLexAop2-IVS-GCaMP6s-p10 50.641 in VK5	this paper	
	w[1118]; P{y[+t7.7] w[+mC]=13XLexAop2-IVS-GCaMP6s-p10}su(Hw)attP1	Bloomington	44274
	w-; GMR-55C05-LexA (attP40)	Bloomington	54897
	w-; GMR-55C05-LexA (attP40); 20xUAS-CsChrimson-mCherry-trafficked in su(Hw)attP1 (3015633), 13xLexAop2-IVS-GCaMP6s-p10 50.641 in VK5	this paper	
	w-; GMR-55C05-LexA (attP40); LexAop-GCaMP6s, UAS-TNT	this paper	
	w-; GMR-38H09-LexA (attP40); LexAop-GCaMP6s, UAS-TNT	this paper	
	w[1118]; TI{w[+mC]=lexA::p65}NPF[lexA]/TM6B, Tb[1]	Bloomington	83720
	w; 60E02-LexA; UAS-Chrimson-mCherry, LexAop GCAMP6s	this paper	
	w[1118]; P{y[+t7.7] w[+mC]=GMR60E02-lexA}attP40	Bloomington	54905
	20B01-LexAp65 (JK22c); 20xUAS-CsChrimson-mCherry-trafficked in su(Hw)attP1 (3015633), 13xLexAop2-IVS-GCaMP6s-p10 50.641 in VK5	Jovanic et al, 2016	
	w*; UAS-GCaMP6s, LexAopjRGeco1a ; sNPFR-T2AGal4	this paper	
	w-;sNPF-R/TM6-Tb(T2A-GAL4)	Bloomington	84691
	pGP-JFRC7-20XUAS-IVS-GCaMP6s 15.641 in attP40	Bloomington	42746
	w[*]; P{y[+t7.7] w[+mC]=13XLexAop2-IVS-NES-jRGECO1a-p10}su(Hw)attP5	Bloomington	64426

Supplementary Method 1 Resource Table

	UAS-GCamP6s, LexAopjRGeco1a ; TI{2A-lexA::GAD}NPFR[2A-AC.lexA]	this paper	
	w[*];; TI{2A-lexA::GAD}NPFR[2A-AC.lexA]	Bloomington	84423
	w[1118]; P{y[+t7.7] w[+mC]=GMR55C05-GAL4}attP2	Bloomington	39106
	w[1118]; P{y[+t7.7] w[+mC]=GMR22E09-GAL4}attP2	Bloomington	49874
	w-; ; UAS-GCamp6s, 22E09-Gal4/TM6, Tb, Sb	this paper	
	w-;;UAS-GCaMP6s (VK0005), LexAop-TNT/TM3,Sb	this paper	
	w*; 55C05-LexA (AttP40); 22E09-Gal4 (AttP2)	this paper	
	w*; 38H09-LexA (AttP40), 13xLexAop2-IVS-GCaMP6s (AttP5)/CyO; UAS-TNT/TM3,Sb	this paper	
	pGP-JFRC7-20XUAS-IVS-GCaMP6s 15.641 in attP40 ; TI{w[+mC]=lexA::p65}NPF[lexA], P{y[+t7.7] w[+mC]=GMR22E09-GAL4}attP2	this paper	
	w*; UAS-GCaMP6s [w+] (attP40) ; UAS-sNPFR RNAi JF02657 [y+] (attP2)	this paper	
	w[*]; P{w[+mC]=UAS-TeTxLC.tnt}R3	Bloomington	28997
	20xUAS-CsChrimson-mCherry-trafficked in su(Hw)attP1	Franconville et. al 2018	
	y*w*;; UAS-RNAi sNPFR JF02657 in attP2	Bloomington	27507
	w[1118]; P{y[+t7.7] w[+mC]=13XLexAop2-IVS-GCaMP6s-SV40}su(Hw)attP5	Bloomington	44589
	w-;; LexAop-GCaMP6s (su(Hw)attP1), UAS-TNT	this paper	
	y,w[1118];P{attP,y[+],w[3`]RNAi-TK [VIE-260B]] (II)	VDRC	60100

Supplementary Method 1 Resource Table

**Antibodies**					
Primary antibodies					
species, target	Supplier	Catalog number / clone name	Dilution	Lot number	Antibody validation
chicken anti-GFP	Invitrogen	A10262	1/1000	2156242	the antibody has been validated by detection of different targets fused to GFP tag in transiently transfected lysates tested as described by the supplier (https://www.thermofisher.com/antibody/product/GFP-Antibody-Polyclonal/A10262).
mouse anti-CHAT	DSHB	ChAT4B1-c (concentrate)	1/50	1ea12/6/18	the antibody has been validated as described by Salvaterra (Neuroscience research, 1996).
rabbit anti-GABA	SIGMA	A2052	1/500	029M4830V	expression of GABA was detected in neucortical cells from the brains of E19 day old rat embyros as described by the supplier (https://www.sigmaaldrich.com/FR/fr/product/sigma/a2052).
rabbit anti-DVGLUT	gift from Hermann Aberle		1/500		the antibody has been validated as described by Mahr and Aberle (Gene Expr. Patterns, 2006).
rabbit anti-mCherry	Biovision (abcam)	5993-100	1/4000	.	The antibody was tested by Western blot aanalysis as described by the supplier
rabbit anti-DsRed	Clontech	632496	1/500	1509043	the quality and performance of the antibody was tested by Western blot analysis as described by the supplier (https://www.takarabio.com/documents/Certificate%20of%20Analysis/632496/632496-101717.pdf?srsltid=AfmBOooVitSlvN_fdUhMDUNSwM6xA0yTDRrTR5Egbaomb9X5qJcIZrp7)

Supplementary Method 1 Resource Table

Secondary antibodies					
species, target, coupled-fluorophore	Supplier	Catalog number	Dilution	Lot number	
goat anti-chicken AlexA Fluor 488	Abcam	ab150169	1/1000	GR3234906-2	
goat anti-mouse Alexa Fluor 647	Jackson Immuno Research	115-605-003	1/200	145779	
goat anti-rabbit Alexa Fluor 647	Jackson Immuno Research	111-605-003	1/200	146340	
goat anti-rabbit Cy3	Jackson Immuno Research	111-165-144	1/500	163657	
**Softwares and algorithms**					
**Name**	**Source**	**Identifier**			
MWT (multiworm tracker) Swierczek et al., 2011	http://sourceforge.net/projects/mwt	Swierczek et al., 2011			
MATLAB R2021a	http://www.mathworks.org				
Fiji 1.54f	http://fiji.sc/				
